# Technical Recommendations for Real-Time Echocardiography and Fluoroscopy Imaging Fusion in Catheter-Based Mitral Valve Paravalvular Leak and Other Procedures

**DOI:** 10.3390/jcm11051328

**Published:** 2022-02-28

**Authors:** Aleksejus Zorinas, Diana Zakarkaitė, Vilius Janušauskas, Donatas Austys, Lina Puodžiukaitė, Gitana Zuozienė, Robertas Stasys Samalavičius, Ieva Jovaišienė, Giedrius Davidavičius, Kęstutis Ručinskas, Eustaquio Maria Onorato

**Affiliations:** 1 Clinic of Cardiovascular Diseases, Institute of Clinical Medicine, Faculty of Medicine, Vilnius University, Santariskiu 2, LT-08661 Vilnius, Lithuania; diana.zakarkaite@santa.lt (D.Z.); vilius.janusauskas@santa.lt (V.J.); lina.puodziukaite@santa.lt (L.P.); gitana.zuoziene@santa.lt (G.Z.); giedrius.davidavicius@santa.lt (G.D.); kestutis.rucinskas@santa.lt (K.R.); 2 Department of Public Health, Institute of Health Sciences, Faculty of Medicine, Vilnius University, M. K. Čiurlionio 21/27, LT-03101 Vilnius, Lithuania; donatas.austys@mf.vu.lt; 3 Clinic of Emergency Medicine, Institute of Clinical Medicine, Faculty of Medicine, Vilnius University, Santariskiu 2, LT-08661 Vilnius, Lithuania; robertas.samalavicius@santa.lt (R.S.S.); ieva.jovaisiene@santa.lt (I.J.); 4 Centro Cardiologico Monzino, Istituto di Ricovero e Cura a Carattere Scientifico (IRCCS), University School of Milan, Via C. Parea 4, 20138 Milan, Italy; eustaquio.onorato@gmail.com

**Keywords:** paravalvular leak, imaging fusion, echocardiography, fluoroscopy

## Abstract

Widespread catheter-based interventions for structural heart disease have overtaken the treatment of paravalvular leaks (PVL). Multimodality imaging techniques play a crucial role in accurate diagnosis, procedure planning and performance. However, PVL closure is often technically challenging due to the complex anatomy of the defects and their relation to surrounding anatomical structures. The application of echocardiography and fluoroscopy imaging fusion (EFF) may simplify challenging imaginative three-dimensional reconstruction of the intracardiac anatomy and facilitate the procedure. To master new technology, personnel must make cognitive changes, overcome a learning curve, and obtain adequate theoretical knowledge. Main aim of this manuscript is to present basic recommendations for EFF application in practice, alongside, each scenario is supported by technically challenging clinical examples. We may conclude that our manuscript may provide useful information for physicians on EEF application in clinical practice.

## 1. Introduction

A catheter-based treatment of patients with PVL has proven to be a safe and effective treatment option [1]. With the experience gained and the appearance of devices dedicated for PVL treatment, the procedural success of the catheter-based PVL closure was enhanced [2,3]. Unfortunately, due to challenges related to tortuous anatomy, location, and understanding PVL spatial and structural relationships between fluoroscopy and ultrasound imaging, some defects remain untreated [3]. Transoesophageal echocardiography (TEE) is an irreplaceable tool in diagnosing and procedural guidance in structural heart diseases. It delivers an accurate evaluation of the anatomical structures, physiologic performance of the heart and assesses the results of catheter-based procedures [4]. However, interventional procedures for structural heart disease cannot be performed effectively without the information provided by fluoroscopy due to its superior visualization of catheters and implantable devices [5]. EFF images have given medical professionals the ability to observe synthesized visualization of the devices, tools, and soft tissue of intracardiac structures on one screen and in the same picture [6,7,8]. The EchoNavigator^®^-system (Philips Healthcare, Best, The Netherlands) is the first software to merge echocardiographic and fluoroscopic imaging data in real-time (RT) during percutaneous cardiac interventions [9]. This technology has advanced into the catheter-based treatment of different structural heart diseases and PVL. It gained confidence among medical professionals [10,11]. However, little literature is currently present on when and how EFF is helpful in mitral PVL catheter-based closure or similar intracardiac procedures. With over five years of experience at our center, we use EFF technology routinely in catheter-based procedures and found it helpful. Main aim of this this manuscript is to present basic recommendations for EFF application in practice, alongside each EFF scenario is supported by technically challenging clinical examples.

## 2. Materials and Methods

We have overlooked our experience in the catheter-based treatment of the mitral valve pathology, intending to summarize and present detailed systematic recommendations for EFF application in practice. It leads to development of three various scenarios, each is supported by real clinical examples where the EFF modality was of paramount importance for procedural success. All patients presented in this manuscript received a specifically designed and manufactured device for PVL. Procedures were performed with the assistance of the EchoNavigator^®^-system (Philips Healthcare, Best, The Netherlands), cardiac fluoroscopy imaging (Allura Clarity, Philips Healthcare; Best, The Netherlands), and RT three-dimensional (3D) TEE (Phillips Epiq7, Philips Healthcare; Best, The Netherlands). A description of the transapical catheter-based procedure of the mitral PVL has been described previously [11]. In addition, a detailed process of EFF provided by the EchoNavigator^®^-system has been described earlier by several authors [6,7,8,9,11].

### 2.1. General Considerations

In the setting of PVL or other defect closure procedures, an essential purpose of the imaging is to deliver a spatial understanding of the defect location. Applicable benefits from EFF may be delivered when fused imaging reproduces a spatial view of the prosthesis or native valve and the defect. Such fused images should allow the interventionist to perceive the spatial layout between the tools manipulated and the targeted anatomical structure.

### 2.2. Technical Recommendations

In our opinion, the most effective way to deliver spatially understandable visual information on the EFF synthesized images is to use “not overcrowded” with unnecessary information echocardiographic views. Those are the standard echocardiographic images with well-known and understandable landmarks. Moreover, fused images must be without “overshadowing” each other; in most cases, echocardiographic ones aggravate or overshadow fluoroscopy. For this purpose, to find the optimal balance, fine-tuning of the brightness/contrast on both sides have to be performed.

Initially, acquisition of the most accurate TEE images of the defects and the prosthesis must be performed. After, the position of the C-arm is oriented/angulated following obtained ultrasound images. In addition, the software of the EFF technology allows to label any X-ray invisible or desired structure as a reference point with unique anatomical markers and transfer it to fluoroscopy.

### 2.3. EFF Scenarios

#### 2.3.1. Scenario A “Side View of the TEE Probe”

**Figure 1:**
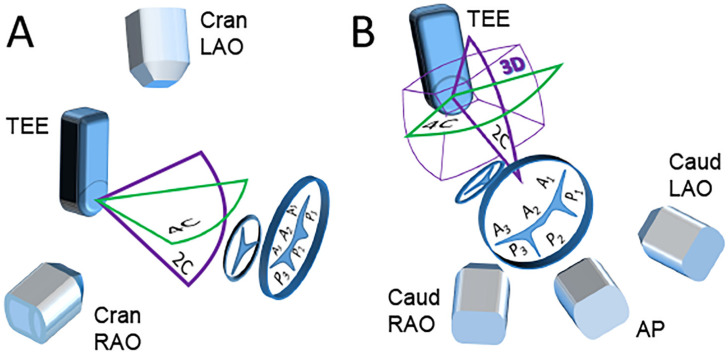
Schematic description of different EFF scenarios. (**A**) “Side view of the TEE probe” scenario. (**B**) “Front view of the TEE probe” scenario. The figure presents standard echocardiography planes (green and purple contour) and three-dimensional imaging volume (3D) with X-ray directions depending on different C-arm projections and mitral and aortic annuli with the segmentation of the mitral valve according to Carpentier [12]. 4C and 2C—four- and two-chamber echocardiography planes, 3D—three-dimensional imaging volume. Positions of the C-arm: Cran—cranial, Caud—caudal, RAO—right anterior oblique, LAO—left anterior oblique, AP—antero-posterior.

In this instance, the TEE probe on fluoroscopy is visible from the side (Figure 1A). Then X-ray beams are more or less “perpendicular” to ultrasound planes. In such TEE probe and C-arm orientation, the most efficient merging occurs between fluoroscopic images with standard, two-dimensional TEE four-, two- and tree-chamber views. Such a scenario applies to the defects whose location, course, anatomy, and regurgitant flow with Color Doppler are well visible in standard TEE views. The C-arm at the cranial and right anterior oblique position allows fluoroscopic observation of the prosthesis from the side. Medially or laterally PVLs will be located on the top or the bottom of the prosthetic ring view without overlapping them both. Such mitral PVLs are visible on two-, four-chamber, or commissural views and optimally fused with fluoroscopic images. When PVLs location is anterior or posterior, they will be visible in three-chamber planes. Optimal projection to fluoroscopic imaging is achieved when the C-arm is at cranial left anterior oblique position.

An example of scenario A: Closure of two mitral PVLs.

**Figure 2:**
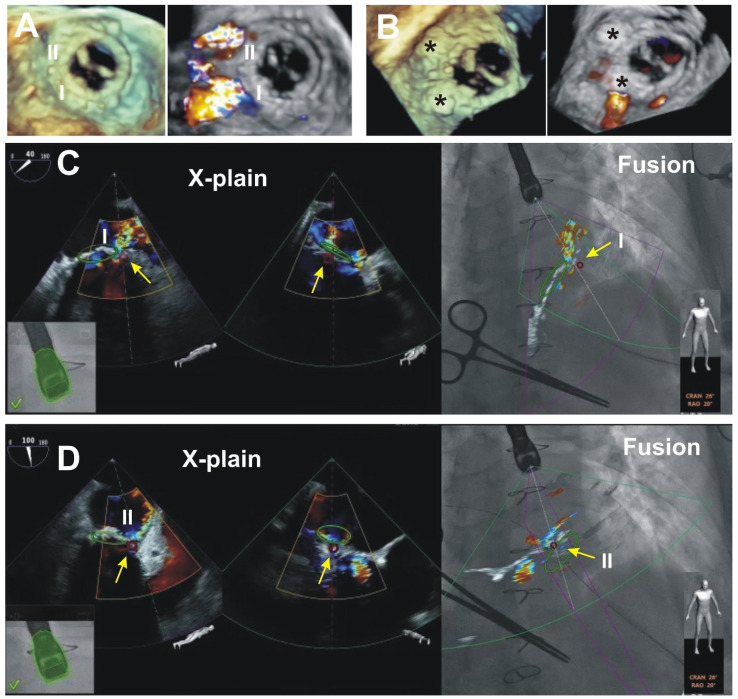
An example of EFF scenario A “Side view of the TEE probe”. The position of the C-arm at 26° Cran, and 20° RAO, TEE probe is seen obliquely—from the side and the top. The automatic recognition of the TEE probe by EchoNavigator^®^-system software is highlighted by the green contouring. (**A**,**B**) preprocedural and postprocedural 3D and 3D Color Doppler TEE images of the biological prosthesis from the surgical perspective. I and II—paravalvular regurgitant jets show the location of two PVLs at 8 and 10 o’clock (**A**). Black stars mark PVL occluders with a small residual regurgitant jet near the first occluder (**B**). (**C**,**D**) intraprocedural EFF images for the closure of the I and II PVLs. Two X-plain (perpendicular to each other) 2D TEE views with Color Doppler cross the I and the II PVLs and regurgitant jets on the left. 2D TEE views planes seen on EFF images tilted in space. In addition, the X-plain views used to construct the markers transferred onto the EFF images (on the right). Green circle—sewing ring of X-ray negative biological valve prosthesis, small red ring (yellow arrows) placed at the PVLs.

A patient presented with symptoms of heart failure (HF). TEE showed a presence of two mitral PVLs: at eight and 10 o’clock, the dimensions of the defects were 4 mm × 4 mm and 4 mm × 3 mm, respectively. An uneventful catheter-based procedure was performed. The PVL at 8 o’clock was closed with 61PLD06W (square with a waist diameter of 6 mm), at 10 o’clock—with 60PLD04W (square with a waist diameter of 4 mm). The residual mitral PVL regurgitation was mild due to near device flow through the defect at 8 o’clock. Therefore, we decided that the result was acceptable, and the procedure was terminated. Technical details of the EFF in this scenario are presented in Figure 2.

#### 2.3.2. Scenario B “Front View of the TEE Probe”

In this scenario, the C-arm is positioned at the angles when fluoroscopy is “facing” TEE probe—caudal left anterior oblique, caudal right anterior oblique, or anterior-posterior orientations (Figure 1B). The X-ray and ultrasound beams are more or less “parallel” towards each other. This rules out standard TEE views projection to fluoroscopy. In this case, an adequate EFF is achieved with 3D TEE views or reconstructions. In this scenario, EFF generates the images “facing” the prosthesis from the ventricular perspective and allows the perception of spatial orientation of PVLs at any location. 

An example of scenario B: Multiple mitral PVL closure.

**Figure 3:**
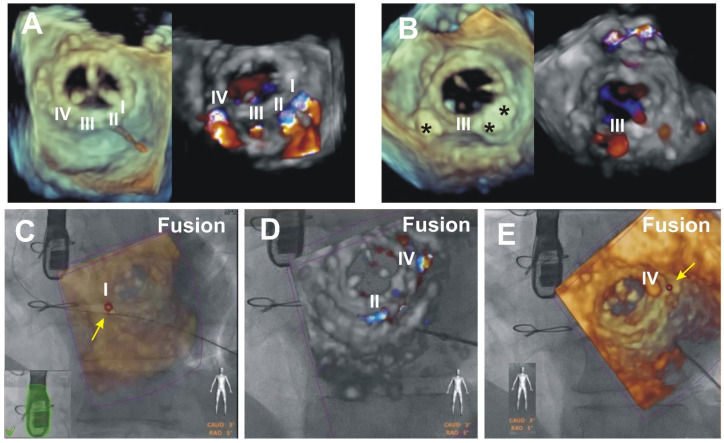
EFF scenario “Front view of the TEE probe”. (**A**) panel—Preprocedural 3D TEE images of the biological mitral prosthesis from the surgical perspective (left). Four (I, II, III, and IV) regurgitant jets through the PVLs seen from 4 to 8 o’clock (right). (**B**) panel—Postprocedural 3D TEE images. The occluders are marked by three black stars (left). Trace residual regurgitant jet thru the third (III) PVL is seen on 3D Color Doppler (right). (**C**–**E**) panels—intraprocedural merged 3D TEE and fluoroscopy images with wire passage thru the I (**C** panel), II (**D** panel) and IV (**E** panel) PVLs. The position of the C-arm is at 3° Caud and 5° RAO, producing a front view of the TEE probe. (**C**) panel—EFF with reduced brightness of the 3D TEE for the optimal view of the wire on the fluoroscopy. (**D**) panel—application of 3D Color Doppler for the second (II) and the fourth (IV) defects. The arrows point to the anatomical markers (red circle) applied at the locations of the first (I) and fourth (IV) PVLs (**C**,**E**).

A patient developed HF symptoms six months after elective MVR. TEE showed the presence of four PVLs at the posterior aspect of the mitral valve from 4 to 8 o’clock, overall generating severe MR. The patient underwent elective catheter-based transapical mitral PVL closure. Three out of four PVLs were closed consequently: the first defect was 5 mm × 4 mm in size and was closed with a PLD 60PLD05W (square with a waist diameter of 5 mm), second was 3 mm × 3 mm, was closed with 62PLD05T square, third was of insignificant dimensions and MR, and fourth was 5 mm × 3 mm—closed with 63PLD05T rectangular. Since MR of the third defect was insignificant, it was left intact. The overall procedural result was excellent, and devices did not interfere with the prosthesis. Residual MR was trivial thru the third defect (Figure 3).

#### 2.3.3. Scenario C “Free View of the TEE Probe”

This scenario occurs when echocardiography views and fluoroscopy merging are not optimal or do not deliver additional information. In this scenario, only anatomical markers labeled at the defect and prosthesis (or native valve annulus, as is in our example) are transferred to EFF view.

An example of scenario C: Defect closure in the anterior leaflet of the mitral valve (AML).

**Figure 4:**
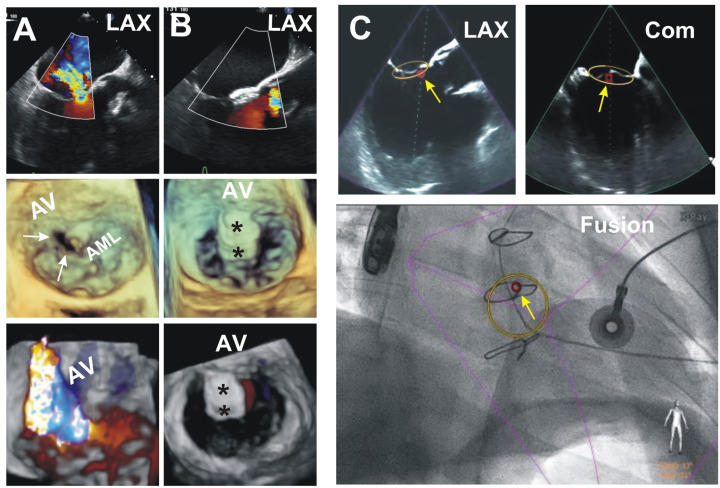
EFF scenario “Free view of the TEE probe”. This figure represents the closure of the two defects in the native anterior mitral valve leaflet (AML). C-arm positioned at 17° Caud and 21° RAO. (**A**,**B**) panels—pre- and postprocedural views of the mitral valve in the long axis (LAX) TEE view with Color Doppler (top). 3D TEE “surgical view” (middle). 3D Color Doppler TEE (bottom). I and II—two defects in the AML. White arrows point to the defects. Black stars—the occluders in the AML. (**C**) panel (top)—long axis view (LAX) and commissural views (Com) perpendicular to each other. Software use labeled mitral annulus with anatomical marker—yellow circle and location of the defects—small red circle. Yellow arrows point to the defects. (**C**) panel, bottom—anatomical markers transferred by EFF software onto fluoroscopy screen.

A patient had presented HF symptoms. A year before, the patient had undergone an AVR with bioprosthesis due to infective endocarditis. At the time of the operation, a large (15 mm) vegetation was found on the aortic valve’s non-coronary cusp. TEE showed severe MR due to two ruptured aneurysms at the A2 aspect of the AML not folding into the leaflets’ coaptation, and no evidence of infective process. Following a heart-team discussion, a decision to close the defect utilizing a catheter-based transapical approach was offered to the patient, which he accepted. The diameter of the perforations was 7 mm in one aneurysm and 4 mm × 2 mm in the other one. Defects were closed by subsequent implantation of the two Occlutech^®^ Paravalvular Leak Devices (Occlutech, Jena, Germany) (PLD), a 60PLD07W (square with a waist of 7 mm) and a 61PLD04W (square with a waist size of 4 mm) with no residual regurgitation thru the defects (Figure 4).

## 3. Results (Outcomes of the Patients Presented)

The patient with two PVLs developed a postprocedural worsening of anemia, requiring two units of packed red blood cell transfusions weekly. The cause for his worsening anemia was mild residual regurgitation thru the PVL at 8 o*’*clock. He underwent a redo MVR 6 weeks later, eventually with a reoccurrence of mitral PVL at 8 o*’*clock. Three weeks after repeat MVR, the patient underwent another catheter-based mitral PVL closure and was discharged home after a challenging course of treatment. Symptoms improved with no need for further blood transfusions. Unfortunately, a year later, due to warfarin overdose, he suffered from a massive hemorrhagic cerebrovascular accident which was lethal.

The patient with the defects in AML and the one with four PVLs under Clinical Trial Principles and Endpoint Definitions for Paravalvular Leaks in Surgical Prosthesis achieved technical, device, procedural, and individual patient success at early and late follow-up [13].

## 4. Discussion

A long-term result in the treatment of mitral regurgitation (MR) depends on the effectiveness of the procedures, meaning that the less residual regurgitation, the better the outcome. Such results are presented in the experiences with various etiology of MR and PVL [14,15]. In PVL treatment, many factors impact early results and long-term outcomes. Those aspects are related to the PVL itself, such as location, shape, anatomy, presence of foreign material, and calcium [16]. In addition, the quality of the imaging performed to diagnose and guide the procedure, complex spatial understanding of the 3D inner anatomy, and adequacy of information shared and interpreted between the interventionist and echocardiography specialists may impact the result [17]. 

A technology of EFF was designed to help guidance of catheter-based procedures. Several years ago, EchoNavigator^®^-system technology that provides live merging of fluoroscopy and 3D TEE was introduced globally, and in 2013, Royal Philips Electronics announced that it had received approval for clinical use by the US Food and Drug Administration. Since then, it has gained global dissemination across a multitude of catheter laboratories. Nowadays, EFF is widely used in the catheter-based treatment of various structural heart diseases, including PVL closure [18]. 

Despite the absence of randomized trials where procedural and long-term results compared between the treatment modalities with and without EchoNavigator^®^-system, this technology may improve treatment results and long-term survival. This manuscript presented three various EFF scenarios supported by several successful examples with complicated geometry or challenging spatial location of various defects related to the mitral valve.

In a patient with two PVLs, a procedure was not challenging and potentially could be performed without EFF technology. This example is presented to provide visual support for scenario A “Side view of the TEE probe’’.

In a patient with multiple PVLs, a demanding part of the procedure was the proximity of PVLs and the size of the relatively small defects for the “wire crossing” target. These were the challenges to overcome during the step-by-step closure of the PVLs in a single procedure. For consequent closure and accurate crossing of the desired PVL, an EchoNavigator^®^-system was employed. Each PVL we planned to close was marked, fluoroscopic and 3D TEE images with labeled leaks overlapped. This software maneuver was of immense help to perform closure in a consequent fashion as was planned. 

In a patient with defects in AML, there were two defects in the native mitral valve leaflet. They were in a constantly moving AML. In addition, difficulty in understanding inner spatial dynamic anatomy on fluoroscopy images was due to the absence of a fluoroscopically visible artificial marker on a mitral valve (such as an annular stent of the prosthesis or other). These factors have created a procedural challenge—crossing the defects with two separate wires. For this stage of the operation, defects were identified and labeled on a TEE image by two markers. The EchoNavigator^®^-system software retranslated the markers in RT onto the fluoroscopic image screen, precisely indicating the fluoroscopic orientation of the leaks. Overlapping the echocardiography view on the fluoroscopic screen allowed an operator to select the best fluoroscopic orientation that helped introduce the wires. 

Our presented examples demonstrated the usefulness of the EchoNavigator^®^-system in various EFF scenarios, including multiple PVLs and defects in the native leaflet of the mitral. We believe and other authors have shown that this technology can reduce procedural time and radiation exposure due to a better understanding of anatomical structures and the spatial relation between the fluoroscopic and ultrasound images [19]. In addition, Balzer et al. have stated that this system can increase the safety, accuracy, and effectiveness of percutaneous interventions of structural heart disease, particularly during PVL closure, facilitating the leak’s location and giving additional information on the surrounding structures [20].

## 5. Limitations

Our presented insights are limited to retrospective observation by the experience at a single center. The inclusion of examples in this manuscript was not standardized prospectively, but rather chosen retrospectively. Therefore, there is a theoretical bias associated with such an investigation; thus, it may not be applicable at other centers.

## 6. Conclusions

Our presented insights may be useful for the physicians starting to or already working with EFF technology. Additionally, our described scenarios may help improve communication between echocardiographers and interventionists, guiding catheter-based interventional cardiac procedures for mitral PVL or other defects. EFF technology facilitates catheter and guidewire pass-through leaks by target markers, allowing assessment of the relationship between the devices and the surrounding structures. Furthermore, EFF may be of paramount importance to guide the wire through the lesion, particularly in the case of invisible biological prosthetic valves or native cardiac structures.
